# Association between clinical and surgical variables with postoperative outcomes in patients treated for intestinal obstruction for non-malignant conditions: a cross-sectional study

**DOI:** 10.1038/s41598-023-41328-6

**Published:** 2023-09-04

**Authors:** Felipe Girón, Carlos Eduardo Rey Chaves, Lina Rodríguez, Roberto Javier Rueda-Esteban, Ricardo E. Núñez-Rocha, Juan Daniel Pedraza, Danny Conde, Marco Vanegas, Ricardo Nassar, Gabriel Herrera, Juan David Hernández

**Affiliations:** 1 Department of Surgery, Fundación Santa Fé de Bogotá, Carrera 7 # 117 - 15, 111711 Bogotá, D.C, Colombia; 2 School of Medicine, Universidad del Rosario, 111711 Bogotá D.C, Colombia; 3 School of Medicine, Universidad de los Andes, 111711 Bogotá D.C, Colombia

**Keywords:** Medical research, Outcomes research, Gastrointestinal diseases, Intestinal diseases

## Abstract

Intestinal obstruction is considered a frequent surgical pathology related to previous surgical procedures. Many different factors can lead to different outcomes when surgical management is needed. Therefore, we aim to describe the factors related to morbidity and mortality in surgical management of IO in a single-center experience. Retrospective observational study with a prospective database, in which we described patients who underwent surgical management due to intestinal obstruction between 2004 and 2015. Demographics, perioperative data, surgical outcomes, morbidity, and mortality were described. 366 patients were included. Female were 54.6%. Mean age was 61.26. Laparoscopic approach was done in 21.8% and the conversion rate was 17.2%. Intestinal resection was performed in 37.9% of the cases. Postoperative complications were observed in 18.85%. Reintervention and mortality were 9.5% and 4.1% respectively. Laparoscopic approach shows lesser time of intestinal transit (mean 28.67 vs. mean 41.95 h), and restart of oral intake after surgery (mean 96.06 vs. mean 119.65) compared with open approach. Increased heart rate and intensive care unit length of stay were related with mortality (*p* 0.01 and 0.000 respectively). For morbidity, laparotomy and need and duration of ICU stay were related with any complication statistically significant (*p* 0.02, 0.008, 0.000 respectively). Patients with increased heart rate in the emergency room, decreased amount of intravenous fluids, need and higher length of stay in the intensive care unit, and delay in resuming oral intake after surgery appear to have poor outcomes. Laparoscopic approach seems to be a safe and feasible approach for intestinal obstruction in selected patients.

## Introduction

Intestinal obstruction (IO) is the restriction of the intestinal content flow at any location of the gastrointestinal tract causing proximal intestinal dilation due to luminal accumulation of air and feces^1^. If not solved, intestinal blood flow could be compromised, leading to ischemia, perforation, and in severe cases, death^1^. IO is one of the most common surgical entities, corresponding to 3% of the surgical emergency admissions worldwide and 12–16% in the United States^2,3^. Abdominal pain, vomiting, abdominal distention, and constipation are the most frequent clinical features^1–3^. However, sometimes clinical presentation is not enough to establish the diagnosis, and plain abdominal radiography or computed tomography (CT) are required. The former showing hydro-aerial levels and the absence of distal gas, and the latter shows a specific site, grade, and etiology of the obstruction^3,4^. Establishing IO-specific location and etiology is a cornerstone for defining the appropriate management. Usually, IO is small bowel related, representing 70–80% of the IO cases, being adhesions the most frequent etiology^2,4,5^. Notwithstanding, hernias, malignancy, and volvulus are also known as frequent etiologies (70%), these could vary in each country, being hernia the most prevalent cause in some developing countries^5^.

The distinction between non-surgical and surgical management should be done, in suspected cases of complicated IO (fever, leukocytosis, high acid lactic level, constant and out of proportion abdominal pain, signs of acute abdomen, among others) foreign body, neoplasm etiology, and/or radiological features of bowel strangulation or perforation, an emergent surgical approach should be performed^6^. Otherwise, conservative management might be preferred based on intravenous fluid rehydration, analgesia, oral intake suspension, and nasogastric tube placement. According to some authors such as Long et al.^6^ 43–73% of the partial and 25–45% of complete bowel obstructions, were solved with conservative management in the absence of clinical signs of complication^6^. At the same time, this approach avoids the inherent risks of surgery, especially in high-risk patients^7^.

IO can be considered a frequent pathology associated with elevated in-hospital costs and mortality rates that could vary between 3 and 7,1%^4,8–10^. Although some clinical and surgical features have been studied, there is still a lack of evidence regarding the relationship between patient perioperative characteristics with postoperative outcomes in patients with non-malignant conditions. Reaching this association could help physicians to promptly determine high-risk patients and carry out early surgery or focus clinical efforts. Therefore, we present the experience of a reference center in the surgical management of IO and the relationship between clinical perioperative variables with postoperative outcomes.

## Methods

With the Institutional Review Board’s approval (Fundación Santa Fe de Bogotá) and following Health Insurance Portability and Accountability Act (HIPAA) guidelines, a retrospective review of a prospectively collected database was conducted. All experimental protocols were approved by a named institutional and/or licensing committee. All patients over 18 years of age who underwent surgical management due to intestinal obstruction between January 2004 and December 2015 were included. Patients with malignant conditions and missing data were excluded. All patients were treated in a high-volume center and following Bologna guidelines. Ethical compliance with the Helsinki Declaration, current legislation on research Res. 008430-993 and Res. 2378-2008 (Colombia), and the International Committee of Medical Journal Editors (ICMJE) were ensured under our Ethics and Research Institutional Committee (Fundación Santa Fe de Bogotá) approval. Demographics, perioperative data, and surgical outcomes were included. 30 days follow-up morbidity and mortality were evaluated.

### Statistical analysis

Descriptive statistics were reported according to the variable nature and distribution. Qualitative analysis was performed in terms of frequencies and percentages, while quantitative analysis was done in terms of mean and standard deviations of normally distributed data and medians and interquartile ranges (IQRs) for non-normally distributed data. Qualitative variables were analyzed using Chi-square statistics (Fisher’s exact test when appropriate). Quantitative variables were analyzed, based on normality, with Spearman’s or Pearson’s associations correlation coefficients accordingly. For associations between categorical variables exact logistic regression was performed, odds ratios with 95% confidence intervals were provided when appropriate, and a statistical value of *p* < 0.05 was defined as the cut-off point. For associations between continuous and categorical variables, two-sided t-tests or U test of Mann–Whitney were performed.

### Ethical approval

Following approval by our institutional review board (Fundación Santa Fe de Bogotá), all procedures performed in this study obeyed the national and Institutional Review Board standards at Fundación Santa Fé de Bogotá. Also, complied with the specified in the Helsinki Declaration of 1964 and succeeding amendments. Informed consent was acquired from all individuals included in the investigation. All experimental protocols were approved by a named institutional and/or licensing committee. Written informed consent was obtained from all individuals included for the publication of this manuscript and accompanying figures.

## Results

### Demographic and previous history characteristics

**Figure 1:**
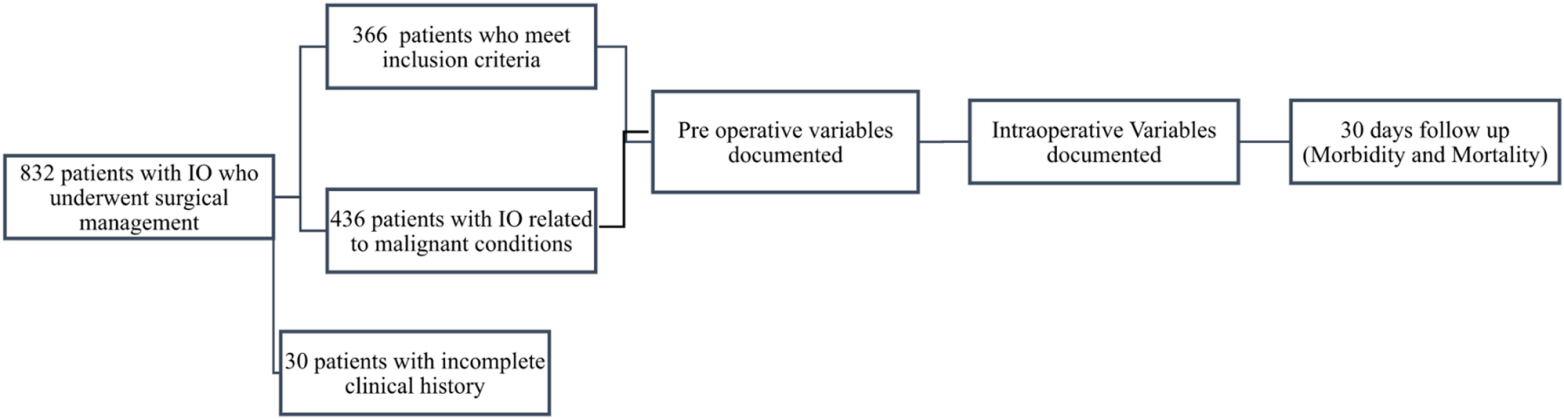
Methodological assessment of the population and the outcomes evaluated.

A total of 366 patients were included in the analysis (Fig. 1). Female patients constituted 54.6% (n = 200) of the population; mean age was 61.26 ± 17.2 years old. Abdominal pain was the most frequent symptom (74.86%), followed by abdominal distension (9.29%). In most of the cases (65.30%) patients presented with at least one comorbidity to the emergency room (ER). Previous history of abdominal surgery was present in 77.87%, and 22.95% of the cases were treated with a surgical approach in a previous intestinal obstruction episode. Abdominal radiation history was observed in 11.48% of the population (Table 1).

**Table 1:** Demographics and clinical features.

Variable	Result
Gender % (n)	n = 366
Male	45.36 (166)
Female	54.64 (200)
Age mean (SD)	61.25 (17.23)
Clinical Features % (n)	
Abdominal pain	74.86 (274)
Abdominal distention	9.29 (34)
No oral intake	5.74 (21)
Dehydration	2.74 (10)
Thoracic pain	0 (0)
Nausea	5.74 (21)
Dyspepsia	0.27 (1)
Diarrhea	1.09 (4)
Diaphoresis	0.27 (1)
Previous history % (n)	
Abdominal surgery	54.92 (201)
Previous history of intestinal obstruction	22.95 (84)
Radiation history	11.48 (42)

### Clinical and pre-operative characteristics

Mean heart rate and systolic arterial blood pressure at the ER were 86.9 ± 16.03 and 126.5 ± 22.3 respectively. 99.1% of the patients were not febrile at the initial encounter. Median duration of clinical symptoms previous to ER entrance was 54.3 h (IQR 2;24). Intravenously administered fluids were also evaluated; in 100% of the cases ringer lactate was used; median fluids administered were 1847 mL (IQR 50; 1300). The up-front surgery approach was decided in 72.13% of the cases. For patients with initial conservative management, the duration of medical management was in most cases 72 h or less. The time between surgical decision and incision was calculated, and the mean time was 2 ± 3.13 h. Initial serum analysis was evaluated; median leukocyte count was 11.3 (IQR 2.2; 10.3); mean hemoglobin was 14.2 ± 2.4, mean creatinine was 1.03 ± 0.5, and mean ureic nitrogen was 19.1 ± 10.5. Electrolyte analysis was made, serum sodium mean was 133.6 ± 8.4; for potassium was 4.5 ± 1.5. Initial abdominal plain radiographic assessment was performed in 73.77% of the cases, and abdominal computed tomography was performed in 78.4% as a complementary study. The American Society of Anesthesiologists (ASA) score was calculated for all patients. Most of the patients were classified as 3—Emergency (35.79%), followed by 4—Emergency with 21.8% of the patients (Table 2).

**Table 2:** Clinical and pre-operative characteristics.

Variable	Result
Physical examination features mean (SD)	
Heart rate (bpm)	86.94 (16.03)
Systolic blood pressure (mmHg)	126.50 (22.39)
Febrile patient % (n)	0.09 (1)
Clinical Features median (IQR)	
Fluids administered	1847 mL (50;1300)
Duration of clinical symptoms (Hours)	54.3 (2;24)
Up-front surgery % (n)	72.13 (263)
Time between surgical decision and incision mean (SD)	2 h (3.13)
Serum analysis	
White blood cell count median (mil/mm3) (IQR)	11.3 (2.2;10.3)
Hemoglobin levels (g/dL) mean (SD)	14.2 (2.4)
Creatinine levels mean (g/dL) (SD)	1.03 (0.5)
Ureic nitrogen mean (g/dL) (SD)	19.1 (10.5)
American Society of Anesthesiologist score	
ASA I	3.00 (11)
ASA I E	6.28 (23)
ASA II	4.09(15)
ASA II E	13.66(50)
ASA III	8.74(32)
ASA III E	35.79(131)
ASA IV	2.1(8)
ASA IV E	21.85(80)
ASA V	2.45(9)
ASA V E	1.91(7)

### Operative characteristics

The laparoscopic approach was preferred in 21.8% of the patients, with a conversion rate of 17.2%. The mean surgical time was 2.6 ± 1.35 h. Intestinal resection was needed in 37.9%. Intraoperative complications were observed in 10.1% of the cases (Table 3).

**Table 3:** Intraoperative characteristics.

Variable	Results
Surgical approach % (n)	
Open	78.12 (286)
Laparoscopic	21.88 (80)
Conversion rate %	17.2%
Surgical Time—Hours—mean (SD)	2.6 (1.35)
Requirement of intestinal resection % (n)	37.9 (138)
Intraoperative complications %(n)	10.1 (36)
Bleeding > 150 ml	83.3 (30)
Visualized Perforation during dissection	16.7 (6)
Intraoperative findings %(n)	
Adhesions	77.87 (285)
Hernia	22.13 (81)

### Postoperative outcomes

Intensive care unit stay (ICU) was required for 48.8% of the patients, with a mean duration of ICU stay of 2.4 ± 7.16 days. In-hospital stay mean was 16.6 (IQR 2; 11). Mean time between surgery and oral intake restart was 114.5 ± 98.5 h. Mean time between surgical approach and intestinal transit was 39.05 ± 79.3 h. Postoperative complications were observed in 18.85% of the population; 3.8% of the cases presented with enteric fistula, surgical site infection was observed in 9.2% and intra-abdominal collections were identified in 4.92%. Postoperative bleeding was evidenced in 2.1% of the patients. Re-intervention rate was 9.5%, and mortality rate was 4.1%. A two-way t-test was performed to compare the time between surgeon consultation, surgical procedure, and the requirement of intestinal resection. Patients that required intestinal resection, have an increased time between surgical consultation and surgical procedure (mean 2.51 h vs. mean 1.68 h), with a statistically significant value (*p* 0.001).

### Open versus laparoscopic approach

A comparison between surgical approaches was made. A t-test was performed. Intestinal transit after surgery was analyzed between groups, the laparoscopic approach shows a lesser time of intestinal transit compared to the open group (mean 28.67 vs. mean 41.95 h) with no statistical differences (*p* 0.09). Time to restart oral intake was also evaluated, open surgery group showed an increased time to restart oral intake compared to the laparoscopic group (mean 96.06 vs. mean 119.65) with statistical significance (*p* 0.02). The in-hospital stay showed slight differences, open group had an increased hospital length of stay compared with the laparoscopic one (mean 14.5 vs. 16.2) with no statistically significant value (*p* 0.1). Mortality rate was higher between groups (open group = 13 patients vs laparoscopic group = 2 patients), also the morbidity rate was higher in the open group compared with the laparoscopic approach (8 patients vs 61 patients) with a statistically significant value (*p* 0.02, OR 2.44, CI 95% 1.11–5.34).

### Statistical analysis

#### Risk factors for mortality

**Figure 2:**
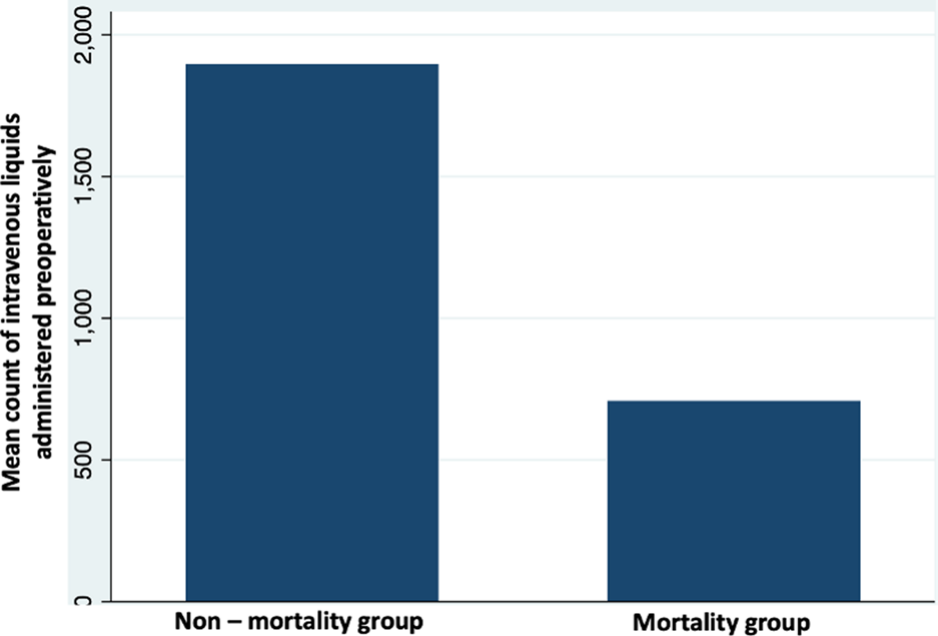
Comparison between mean count of preoperatively administered liquids and mortality.

Logistic regression analysis was performed. Vomiting at the ER has a statistically significant relationship with mortality (*P* 0.05, OR 7.03, CI 95% 0.9–53.1); as well, the presence of abdominal pain shows a p-value of 0.006 with an OR 30.07 (CI 95% 2.5–34.7) in relation with mortality. Laparotomy, ICU requirement, and pulmonary edema were related to mortality with a statistically significant value (p 0.03; 0.01; 0.02 respectively). A T-test was performed to evaluate the relationship between quantitative variables and mortality. Mean comparisons were performed; mortality group patients enter the ER with an increased heart rate (mortality group 94.13 vs non-mortality group 86.63) with a statistically significant value (*p* 0.03). As well, patients in the mortality group have a lesser proportion of administered intravenous fluids at the ER (mortality group 708.66 mL vs non-mortality group 1896.22 mL) with a statistically significant value (*p* 0.002), there is no relationship between time to surgery (< 2 h to surgery) a T-test were performed, showing similar means of intravenous fluids in both groups (1804 mL vs 1870 mL *p* = 0.700) (See Fig. 2). ICU stay in non-mortality group patients was significantly lesser compared with mortality group (1.6 vs. 15.7 days) (*p* 0.000). These factors are related as independent ones to mortality. However, in multivariate analysis, just heart rate and ICU stay duration showed statistically significant values (*p* 0.01 and 0.000 respectively).

#### Risk factors for overall complications

**Figure 3:**
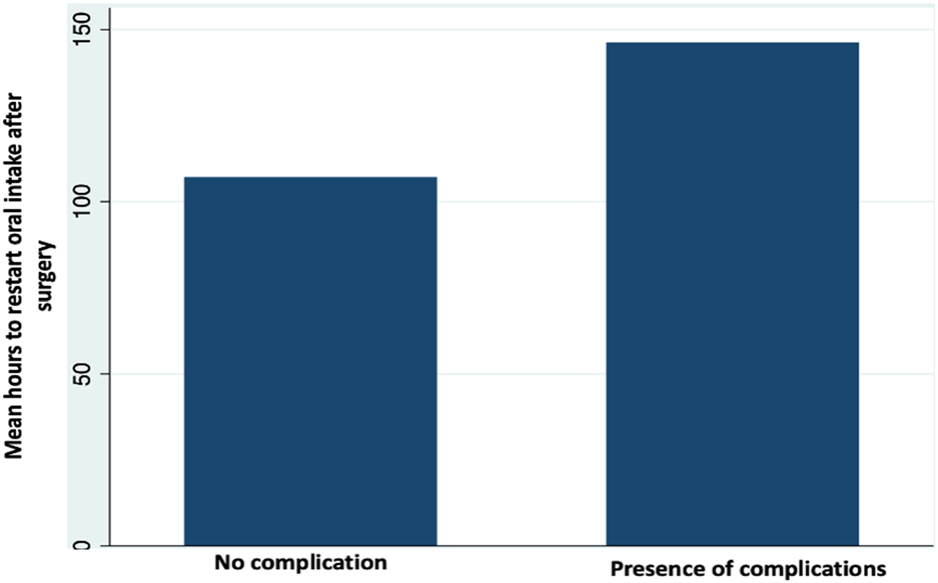
Comparison between mean hours of oral intake after surgery and complications.

Logistic regression analysis was performed. The laparotomy approach showed an OR 4.1 (CI 95% 1.2–12.2) with statistical significance with any complication (*p* 0.009). As well, ICU requirement showed a statistical relationship (*p* 0.00). A T-test was performed to evaluate the relationship between quantitative variables and mortality. Mean comparison was performed. Mean duration of ICU stay in any complication group was higher compared with the no-complications group (1.34 vs. 8.1 days) with a statistical relationship (*p* = 0.000), also mean time between surgical procedure and restart of oral intake was higher in patients who present any complication (107.14 vs. 146.17 h) with a statistically significant value (*p* 0.001, CI 95%) (See Fig. 3). These variables are related as independent factors with any complication. In multivariate analysis; laparotomy, ICU requirement, and duration of ICU stay were related with any complication showing a statistically significant value (*p* 0.02, 0.008, 0.000 respectively).

## Discussion

Acute intestinal obstruction occurs due to the immediate interruption of the flow of intestinal contents along the gastrointestinal tract, which usually represents a common surgical emergency secondary to the appearance of intestinal ischemia that could progress to perforation, peritonitis, or death^1^. Among the causes described for the appearance of this condition are adhesions, hernias, neoplasms, malignancy, and others^11^.

Symptomatology depends on the degree and level at which the respective obstruction is present^12^. The symptoms most commonly associated with IO are usually colicky abdominal pain, nausea, vomiting, absence of flatus, and absence of bowel movements^12^. On physical examination, dehydration, hypotension, abdominal distension, and absence of bowel sounds are usually found^11^. Also, leukocytosis at the expense of neutrophils and band forms, and even the presence of metabolic acidosis may be found in the additional laboratories^11^.

In our study, the main symptoms reported were abdominal pain (74.86%), followed by abdominal distension (9.29%), similar results to those described in the literature^11^. Furthermore, an important factor in the development of IO in our population was that 77.87% of the patients had a history of abdominal surgery and of these, 23% had presented a previous episode of acute IO, with adhesions being a possible etiological diagnosis in these cases^13^. In a systematic review by Rami & Cappell in 2017^11^ adhesions were found to comprise 60–70% of all etiologies of bowel obstruction, followed by 15% secondary to abdominal hernias, 7% due to Crohn's disease, and 5% secondary to small bowel neoplasms, results similar to those found in our population being adhesions (77,87%) and hernias (22,13%) the most frequent causes.

Treatment of IO with a history of abdominal surgical procedures is usually done by non-operative management consisting of nasogastric tube decompression, administration of intravenous fluids, electrolyte replacement, and clinical observation^14^. At the moment, and based on Bologna Guidelines, non-operative management should not be considered for more than 72 h without clinical improvement and resolution of IO. Nevertheless, there is no consensus in terms of surgical approaches such as open or laparoscopic surgery^15^. This has generated controversy since surgery itself is a risk factor for the appearance of new adhesions^14^. However, it is clearly described that surgical management should be chosen in cases in which peritonitis, strangulation, and ischemia are present^15^.

Considering the short range of options between open and laparoscopic approaches, the surgeon must decide based on the patient's current status and experience. The laparoscopic approach has become the main surgical approach of choice for IO^16^. However, some surgeons argue to prefer open surgery since laparoscopy reduces the working space and heightens the risk of iatrogenic lesions^17^. In view of this, it is of utmost importance to mention that laparoscopy should not be used in patients who are hemodynamically unstable but should be used in those with IO that does not resolve in a gastrografin study or in stable patients with high-risk of ischemia^11^.

Due to the design of our study, only those patients who received surgical treatment were included in our population. Of these, 21.8% of the patients were managed by a laparoscopic approach with an average surgical time of 2.6 ± 1.35 h. In addition, bowel resection was required in approximately 38% and an intraoperative complication rate of 10%. Likewise, a conversion rate of 17.2% was found. This is similar to what is found in the literature. Nakanwagi et al.^18^ evaluated 135 patients who required emergency management for IO, showing that 72.7% of the patients required surgical management with bowel resection and anastomosis, a comparable result with our population^18^. Additionally, a study published by Mancini et al.^19^ which sought to compare the laparoscopic approach and open surgery for IO, found that 88.6% of patients required open approach and 11.4% required the laparoscopic approach with a conversion rate of 17.2%. Rate exactly the same as the one found in our population. This evidence shows that the laparoscopic approach seems to be safe and feasible in adequately selected patients^19^.

Regarding postoperative outcomes of surgical management of IO, in our population, a total complication rate of 18.85% was found, being enteric fistulas (3.8%), surgical wound infection (9.2%), and intra-abdominal collections (4.9%) the most frequently presented. Results similar to the previous series where surgical infections have been reported as the most common complications (11.99%), followed by prolonged ileus (9.26%), basal atelectasis, sepsis, and enteric fistulas (1.9%), the latter being similar to our study^20^. Postoperative bleeding rate was 2.1%, comparable results to those reported by Nakamura et al.^21^ where the rate of bleeding was 1%.

On the other hand, mean time between surgery and oral intake restart was 114.5 ± 98.5 h, and mean time between surgery and intestinal transit was 39.05 ± 79.3 h. This is important since it has been proposed that delay in surgical treatment for strangulated IO usually increases the risk of intestinal resection, increased hospital stay, and a significant increase in mortality. Our study shows that an increased time between surgeon consultation and surgical procedure is associated with requirement for intestinal resection (*p* 0.001)^22,23^. Also, the mortality rate was 4.1%, which is relatively low when compared to other studies where total mortality rates have been found to be 17% (16% for open surgery and 1% for laparoscopy)^24^ but similar to that found in studies reported by Soressa et al.^25^.

Along the same lines, a study published by Wullstein and Gross^26^ sought to compare laparoscopic versus open surgical approaches for the acute treatment of small bowel adhesions causing IO and found that major intraoperative complications (bowel movement later than day 6, anastomotic leakage, wound infection, deep vein thrombosis, death) occurred in 15 patients in the laparoscopic group and 8 patients in the open surgery group (*p* 0.156). Likewise, postoperative complications occurred in 19.2% and 40.4% of patients treated with laparoscopic and open surgery respectively. Furthermore, bowel movements started 3.5 days after surgery in the case of laparoscopy and 4.4 days in open surgery (p 0.001)^26^. Also, mean time between surgery and oral intake was 5.1 in the laparoscopy group and 6.4 in the open surgery group (*p* 0.004). Finally, the length of hospital stay was 11.3 for laparoscopic approach and 18.1 in the case of open surgery^26^. Our data shows a favorability of the postoperative outcomes in laparoscopic group patients; with lesser time to intestinal transit and restart of oral intake with a statistically significant value; as well mortality rate was higher in open surgery; however, fails to reach significant value. This data is in line with the one reported in the LASSO trial by Sallinen et al.^27^, in which laparoscopic approach in selected patients shows quicker recovery, as well In terms of morbidity, the laparoscopic approach shows a lesser rate of complications; and according to our results open approach increases the risk in 2.4 times to have any complication with significant statistical value (*p* 0.02), similar data to the one reported by Wullstein et al. and Sallinen et al.^26,27^.

There is still a lack of evidence in terms of the factors related to complications and mortality in patients with non-malignant IO. Some studies tried to elucidate the possible prognostic factors of patients treated with a surgical approach for bowel obstruction. Mariam et al.^28^ show that patients with increased length of hospital stay, illness duration, and presence of any comorbidity, are more likely to have adverse outcomes after a surgical procedure. This is similar to the reported by Derseh et al.^29^, who showed that older patients, with duration of symptoms > 24 h, have poor postoperative outcomes with statistical significance. In our study, increased heart rate at the ER showed a statistical relationship with mortality, there is no evidence in terms of these findings; however, tachycardia could be related to a high grade of dehydration or septic disease. Also, in our population increased ICU stay is related to mortality with statistical significance; this data is similar to the reported by Simmachew et al.^30^, in which increased hospital length shows poor outcomes regarding mortality.

In terms of morbidity, our study demonstrates that open approach is related to postoperative complications; this data is comparable with the one reported by Nordin et al.^31^, who showed that patients treated with laparoscopic approach have reduced hospital length stay, surgical time and postoperative complications compared with an open approach. As well, ICU requirement and duration are related to poorer outcomes regarding the presence of any complication, data that agree with those evidenced by Derseh and Mariam et al.^28,29^. In the independent analysis, our study demonstrates that a lesser rate of intravenous fluids prior to surgery is related to mortality (mortality group 708.66 mL vs. non-mortality group 1896.22 mL) (*p* 0.002); this data suggest that patients with IO will benefit from high-volume replacement regimen to avoid postoperative mortality.

Among the limitations of this study are its retrospective nature, the scarcity of previous studies to compare our results, and also when comparing surgical approaches there may be a selection bias with less compromised patients in the first group. However, the sample size and the standardized treatment based on Bologna guidelines with an experienced group of general surgeons in a high-volume institution are included in the strengths of our study.

## Conclusion

Patients with increased heart rate in the emergency room, less proportion of intravenous fluids administered preoperatively, requirement of ICU, duration of stay at intensive care unit, and delay in the restart of oral intake after surgery seem to have poor outcomes defined as mortality or any complication in patients with intestinal obstruction and should be considered as high-risk cases. Our data is in line with the LASSO trial, and shows that laparoscopic approach in selected population seems to be a safe and feasible approach for patients with intestinal obstruction, with acceptable rates of morbidity and mortality, and with a quicker time of recovery of intestinal transit, and restart of oral intake. Additional prospective studies with larger sample sizes are needed to validate our results.

## Data Availability

The datasets used and/or analyzed during the current study are available from the corresponding author upon reasonable request.
